# A Stress-Induced Martensitic Transformation in Aged Ti_49_Ni_51_ Alloy after High-Velocity Impact

**DOI:** 10.3390/ma9070500

**Published:** 2016-06-23

**Authors:** Yingying Zhu, Haizhen Wang, Zhiyong Gao, Wei Cai

**Affiliations:** Science and Technology on Materials Performance Evaluation in Space Environment Laboratory, School of Materials Science and Engineering, Harbin Institute of Technology, Harbin 150001, China; zhuyingying841225@163.com (Y.Z.); sleepsid@163.com (H.W.); waicai@hit.edu.cn (W.C.)

**Keywords:** aged Ti_49_Ni_51_ alloy, microstructure, martensitic transformation, high-velocity impact, regionalization characteristics

## Abstract

The effects of a high-velocity impact on the microstructure, phase transformation and mechanical property of aged Ti_49_Ni_51_ alloy are investigated. The transformation behavior and microstructure along the impact direction after impact emerge with regionalization characteristics, including a deformed region near the crater (0–4 mm) and an un-deformed region of the distal crater (5–6 mm). Stress-induced martensite is the main deformation mechanism in the deforming region of aged Ti_49_Ni_51_ alloy under high-velocity impact.

## 1. Introduction

TiNi shape memory alloys (SMA) have many current and potential engineering applications since their shape memory and superelasticity properties relate to the martensitic transformation induced by thermal or stress [1,2,3]. In aeronautics and astronautics applications, SMA devices would suffer a high-velocity impact from space debris, which often results in a change in the transformation behavior and microstructures [4,5].

As we know, aging treatment is an effective technology to adjust a material’s performance [6]. The aging of Ni-rich TiNi alloy can lead to the precipitation of Ti_3_Ni_4_, Ti_2_Ni_3_ and TiNi_3_ precipitates. Ti_3_Ni_4_ is coherent with the matrix B2 and is the most influential in affecting the transformation behavior. Unlike fully annealed and quenched near-equiatomic TiNi alloys, which transform from B2 to B19’ directly, aged Ni-rich TiNi alloys normally transform in two stages (B2-R-B19’) [7,8,9].

Liu investigated the shock-induced transformation behavior in NiTi SMA, and a three-step reverse phase transformation was observed [10]. Kurita et al. studied the transformation behavior of shock-compressed Ni_48_Ti_52_; a one-step transformation was observed before the shock treatment, and a three-step transformation was also found after annealing at an appropriate temperature on the shock-treated Ni_48_Ti_52_ [11]. The appearance of a three-step phase transformation may be because the shock wave makes the energy difference between the grain interior and grain boundary increase, and this results in the desynchronization of the phase transformation process.

The above research is conducted on the transformation behavior and microstructures of solution-treated TiNi alloys. So far, the research of high-velocity impact on aged Ti_49_Ni_51_ alloy is still in blank. In this paper, the transformation behavior and deformed microstructures in aged Ti_49_Ni_51_ alloys under high-velocity impact are characterized to investigate the deformed microstructure evolution from the crater rim to the matrix.

## 2. Materials and Methods

Ti_49_Ni_51_ shape memory alloy rod was hot-forged at 1173 K by Beijing Shape Memory Company (Beijing, China). Specimens with 17.5 mm diameter and 12 mm thickness were cut from the rod using a spark-erosion cutting machine. All the sealed samples were solution treated at 1173 K for 2 h and water-quenched to obtain a supersaturated homogeneous solid solution, and then aged at 773 K for 1 h.

**Figure 1:**
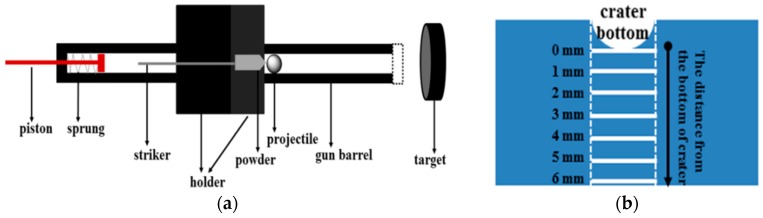
Schematic diagram of the powder gun equipment (**a**) and sampling modes of DSC and TEM specimens (**b**) [13].

Impact experiments were carried out on a powder gun, with a schematic diagram of the powder gun equipment shown in Figure 1a. A GCr15 steel ball with a 3 mm diameter was used as a projectile with the distance between the target and gun barrel being ~10 cm. The impact velocity was measured using a magnetic instrument [12] and a muzzle velocity of ~1.03 km·s^−1^ was obtained.

After impact, aged Ti_49_Ni_51_ alloy specimens were cut in a longitudinal direction using a spark-erosion cutting machine and polished mechanically. As shown in Figure 1b, thin sheets (represented by white lines) on the edge of the crate along the impact direction were cut and examined by differential scanning calorimetry (DSC, Perkin Elmer Diamond, New York, NY, USA) at 20 K·min^−1^ from 373 K to 193 K, the sample was heated from room temperature to 373 K, kept for 1 min, then cooled to 193 K, kept 1 min, and then heated to 373 K. Transmission electron microscopy (TEM) studies were carried out using a Philips CM-12 microscope (FEI, Rotterdam, The Netherlands) at 120 kV to observe the sample microstructure. Vickers hardness measurements were conducted using 300 g loads for a loading time of 15 s using a MMT-3 microhardness tester (Matsuzawa, Tokyo, Japan). Samples used for hardness measurement were ground and polished.

## 3. Results and Discussion

### 3.1. Transformation Behavior

**Figure 2:**
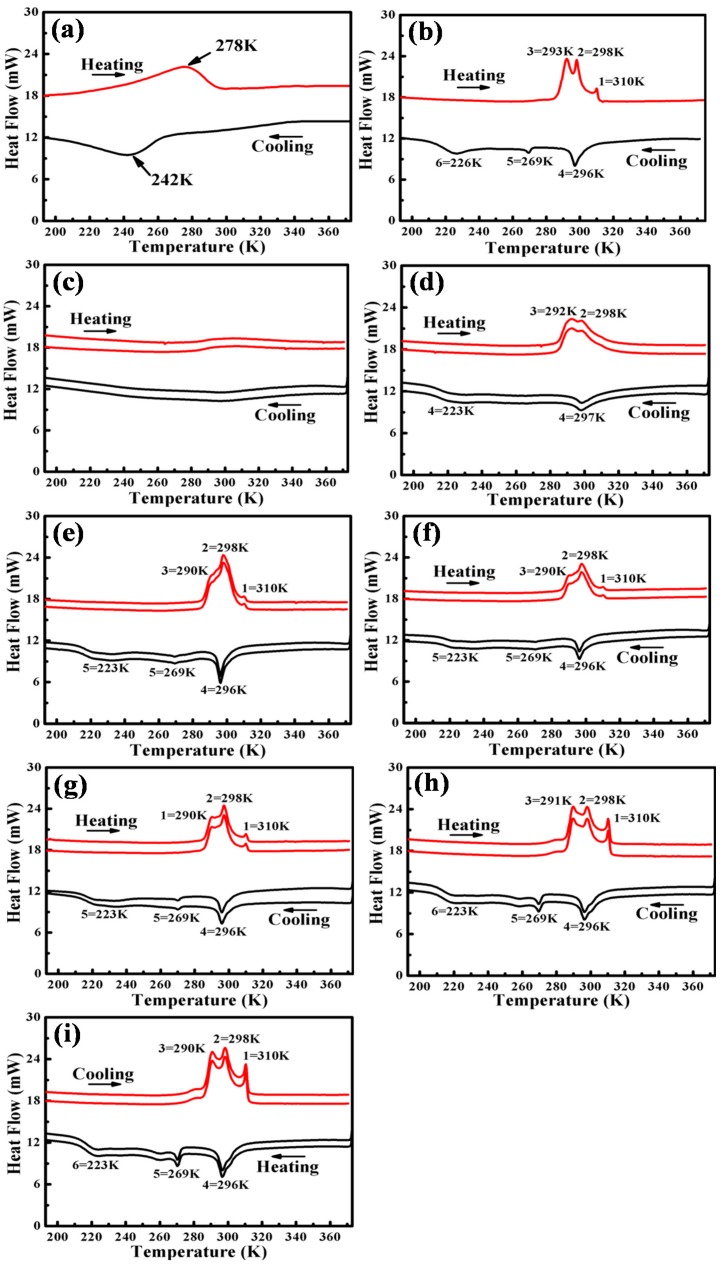
DSC curves for aged Ti_49_Ni_51_ alloy with different distances (0–6 mm) from the crater before and after impact: (**a**) solution-treated; (**b**) non-impact; (**c**) 0 mm; (**d**) 1 mm; (**e**) 2 mm; (**f**) 3 mm; (**g**) 4 mm; (**h**) 5 mm; (**i**) 6 mm.

**Figure 3:**
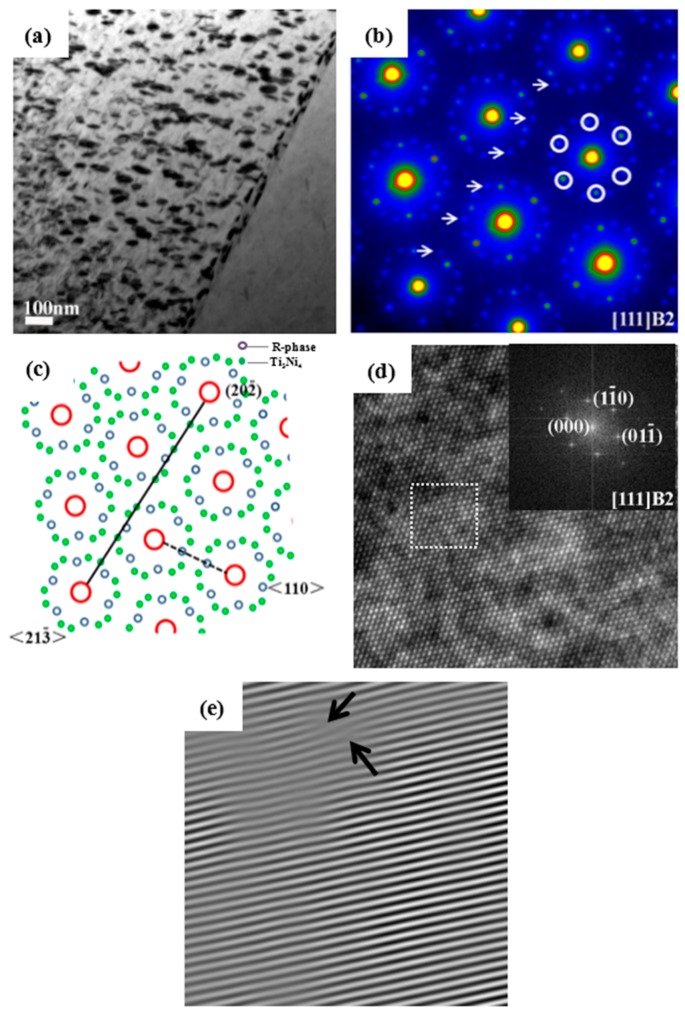
TEM micrographs and SAED patterns of aged Ti_49_Ni_51_ alloy before impact: (**a**) Bright-field image; (**b**) SAED pattern; (**c**) Corresponding schematic representation of (**b**); (**d**) High-resolution TEM image of (**a**); (**e**) Inverse Fourier filtered image of the area indicated by a dotted square in (**d**).

Figure 2 shows the DSC curves of aged Ti_49_Ni_51_ alloy specimens after impact during two thermal cycles with different distances (0–6 mm) from the bottom of the crater. A one-step phase transformation occurs corresponding to B2↔B19’ for solution-treated Ti_49_Ni_51_ alloy in Figure 2a. The DSC curves of the aged Ti_49_Ni_51_ sample before impact are shown in Figure 2b, and it can be seen that multi-step phase transformation occurs compared with the solution-treated Ti_49_Ni_51_ alloy. This clearly shows that peak 5 and peak 1 are a pair of phase transformation. They may show a B19’↔B2 transformation in the grain interior, where Ti_3_Ni_4_ particles are essentially free. The cooling peak 4 corresponds to its reverse transformation peak 2, which occurs in the grain boundary region. As this pair of peaks has only a narrow hysteresis of 2 K, we can deduce that it may be a B2→R transformation and its reverse transformation. From the microstructure of this sample, as shown in Figure 3a, which shows a segregation of Ti_3_Ni_4_ particles around the grain boundaries, we can identify that peak 4 and peak 2 correspond, respectively, to the R phase transformation and its reverse transformation of the grain boundary regions. In addition, peak 6 and peak 3 are a pair of phase transformation, and they may show a R↔B19’ transformation at the grain boundary region, as the R phase transformation occurs only in the presence of Ti_3_Ni_4_ particles [14]. As Fan et al. pointed out, the occurrence of a three-step martensitic transformation for aged TiNi alloy depends on the distribution of Ti_3_Ni_4_ particles between the grain interior and grain boundary [15].

The DSC curves of impacted Ti_49_Ni_51_ alloy are presented in Figure 2c–i. On the bottom of the crater (0 mm), the exothermic and endothermic peaks are too wide to be observed in the range of from 193 K to 373 K. As the distance is 1 mm, two endothermic peaks (peak 2 and peak 3) before impact partially overlap after impact, and this may be because the stress concentrated in the grain boundary during the high-velocity impact, and the stress-induced martensite phase transformation happens while, at the same time, the uneven distribution of stress leads to the width and overlap of peak 2 and peak 3 [16]. The exothermic and endothermic peaks become sharper gradually with the increasing distance (2–4 mm), as shown in Figure 2d–g; meanwhile, peak 3 and peak 2 separated gradually due to less deformation. As the distance is 5–6 mm, the exothermic and endothermic peaks in Figure 2h,i are quite similar to the transformation behavior before impact treatment (Figure 2b). 

### 3.2. Deformed Microstructure

Figure 3 shows the TEM micrographs and the selected area electron diffraction (SAED) pattern of aged Ti_49_Ni_51_ alloy. Aged Ti_49_Ni_51_ alloy exhibits finely dispersed Ti_3_Ni_4_ particles embedded in the grain interior (Figure 3a). The SAED pattern is shown Figure 3b, and it should be noticed that the microstructures are B2 austenite, R phase corresponding to 1/3 <110> B2 super-lattice spots marked with some white circles, and Ti_3_Ni_4_ particles corresponding to 1/7 <213> B2 super-lattice spots marked with some white arrows [17]. The corresponding schematic representation is shown in Figure 3c (blue circles represent the R phase, green circles represent Ti_3_Ni_4_ particles). Moreover, the dislocation density is approximately calculated to be 8.90 × 10^11^ cm^−2^ according to the high-resolution TEM image in Figure 3d,e.

**Figure 4:**
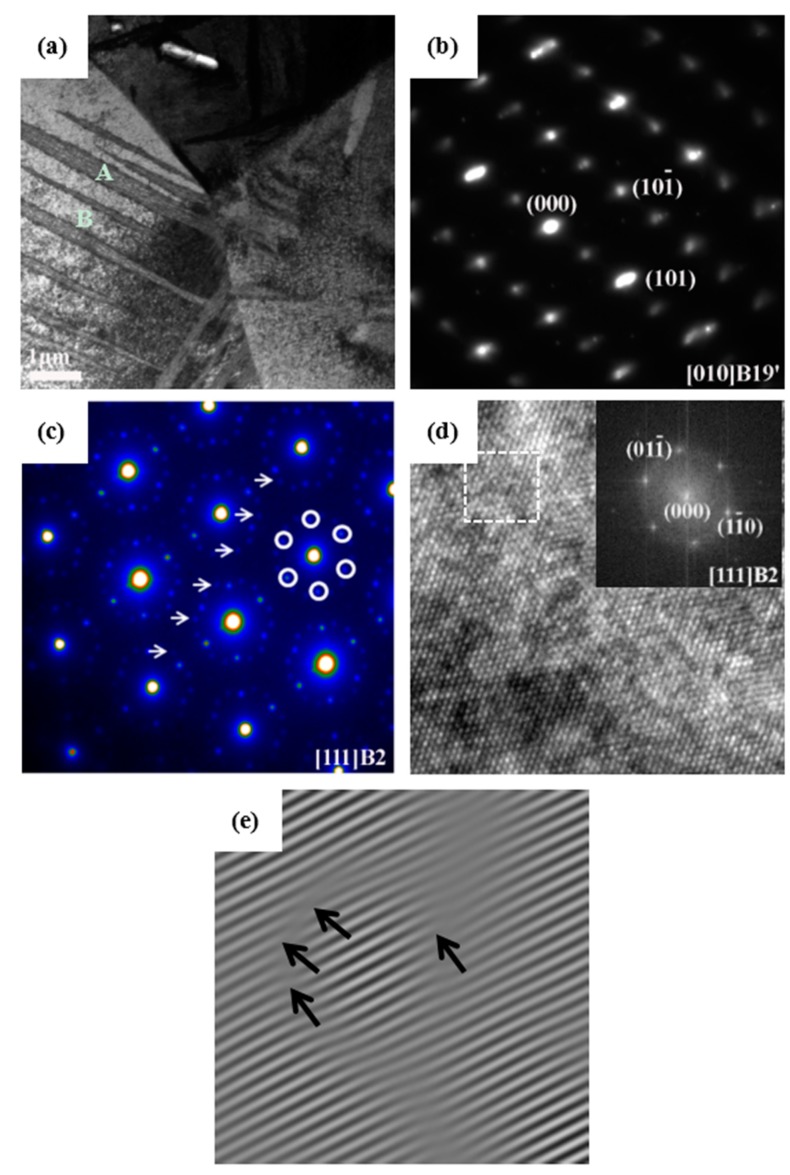
TEM micrographs and SAED patterns of aged Ti_49_Ni_51_ alloy after impact: (**a**) Bright-field image of the bottom of crater (0 mm); (**b**) SAED pattern of area A in (**a**); (**c**) SAED pattern of area B in (**a**); (**d**) High-resolution TEM image of (**a**); (**e**) Inverse Fourier filtered image of the area indicated by a dotted square in (**d**).

The TEM and SAED patterns of Ti_49_Ni_51_ alloys on the bottom (0 mm) of the crater after impact are shown in Figure 4a–c; a lot of plates appear in Figure 4a, and from the SAED pattern in Figure 4b, the structure of the martensite plates is B19’. Meanwhile, the parent phase, R phase, and Ti_3_Ni_4_ particles were found in Figure 4c. According to the high-resolution TEM image in Figure 4d,e, the dislocation density is approximately calculated to be 1.40 × 10^12^ cm^−2^, which is no significant increase compared with of the density before impact.

From Figure 4a, the martensite plate begins to nucleate in the grain boundary region, and this may be because the shock treatment increases the non-chemical free energy such as the strain energy and interfacial energy of the grain boundary, which provide part of the driving force for nucleation and the growth of martensite; as a result, a martensite plate grows from the grain boundary to the grain interior. Partial Ti_3_Ni_4_ particles exist in the interior of the martensite plate.

In conclusion, we can deduce that the deformation mechanism of aged Ti_49_Ni_51_ alloy under high-velocity impact is stress-induced martensite rather than a mixture of stress-induced martensite and dislocation generation. This is different from our previous study of high-velocity impacted Ti_50_Ni_50_ alloy [18]. It may be because the Ti_3_Ni_4_ dispersed uniformly in the matrix and enhanced the strength of the matrix that dislocation is difficult to introduce.

**Figure 5:**
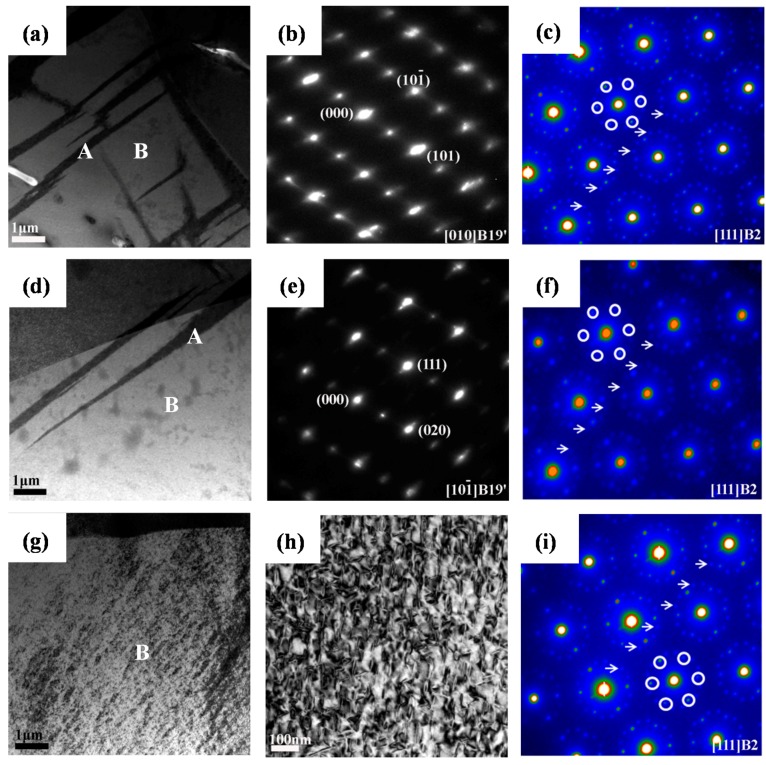
TEM micrographs and SAED patterns of aged Ti_49_Ni_51_ alloy after impact: (**a**) Bright-field image of 1 mm; (**b**) SAED pattern of area A in (**a**); (**c**) SAED pattern of area B in (**a**); (**d**) Bright-field image of 2–4 mm; (**e**) SAED pattern of area A in (**d**); (**f**) SAED pattern of area B in (**d**); (**g**) Bright-field image of 5–6 mm; (**h**) Magnified microstructures of area B in (**g**); (**i**) SAED pattern of area B in (**g**).

As the distance increases from the crater, the volume fraction of the martensite plates reduces due to the decrease in the material stress, strain, strain rate and degree of deformation, as shown in Figure 5a–f. When the distance from the crater is 5–6 mm, the microstructure is similar to that of non-impact. In short, the transformation behavior and microstructure along the impact direction after high-velocity impact emerge with regionalization characteristics; the bottom of the crater was classified into a deformed region near the crater (0–4 mm) and an un-deformed region of the distal crater (5–6 mm).

**Figure 6:**
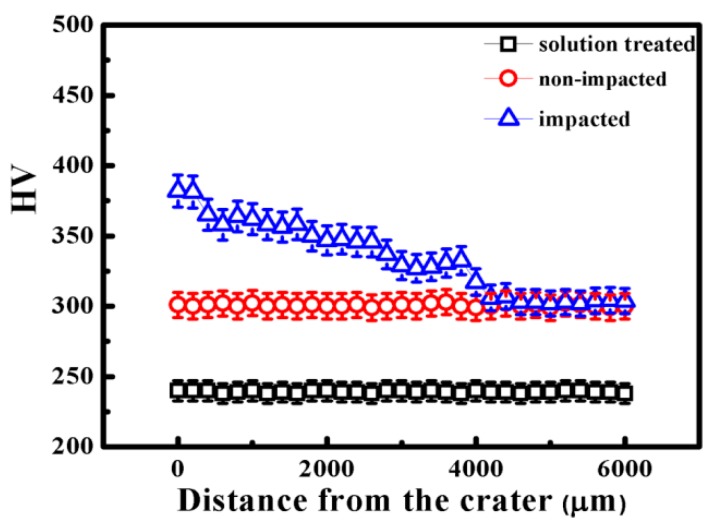
Microhardness distribution along crater in aged Ti_49_Ni_51_ alloy before and after impact.

Figure 6 shows the microhardness of aged Ti_49_Ni_51_ alloy before and after impact. The microhardness for solution-treated Ti_49_Ni_51_ alloy is ~240 under 300 g for 15 s, and the microhardness for aged Ti_49_Ni_51_ alloy before impact is ~300. While the microhardness near the crater increased to ~380, this may be because the material was subjected to the most severe plastic deformation during impact. Moreover, the microhardness decreased with the increasing distance from the crater due to the decrease in the material stress and degree of deformation. The microhardness of 4–6 mm from the crater approximates that of non-impact. Therefore, impact can achieve a gradient variation in the deformation degree from the bottom of the crater to the deep matrix.

## 4. Conclusions

The transformation behavior, microstructure and mechanical property of aged Ti_49_Ni_51_ alloy can achieve a regionalized variation from the bottom of the crater to the deep matrix. The generation of stress-induced martensitic transformation is due to the concentration of stress in the grain boundary, under the shock waves. The small change of dislocation density is because of the enhanced matrix strength due to Ti_3_Ni_4_ particles. The number of martensite plates and the microhardness reduce with the increasing distance from the crater.
